# PCKP versus UPKP for moderate-to-severe osteoporotic vertebral compression fractures: a prospective randomized controlled trial revealing synergistic mechanisms of optimized bone cement distribution and anti-osteoporosis therapy on medium-to-long-term outcomes

**DOI:** 10.3389/fragi.2026.1652951

**Published:** 2026-04-08

**Authors:** Fuguo Yang, Yuanchao Luo, Kang Chen, Renjian He

**Affiliations:** Department of Orthopedics, Zigong First People’s Hospital, Zigong, China

**Keywords:** osteoporotic vertebral compression fractures (OVCFs), percutaneous curved vertebral kyphoplasty (PCKP), unilateral puncture percutaneous kyphoplasty (UPKP), anti-osteoporosis treatment compliance rate, medium- to long-term efficacy

## Abstract

**Background:**

Percutaneous curved kyphoplasty (PCKP) demonstrates early advantages in treating osteoporotic vertebral compression fractures (OVCFs), but its long-term efficacy and patient-selection criteria remain controversial.

**Objective:**

The aim of this study is to prospectively compare the mid-to-long-term outcomes of PCKP versus unilateral percutaneous kyphoplasty (UPKP) for single-level OVCFs and establish a hierarchical decision model based on vertebral compression severity.

**Methods:**

A total of 139 patients with single-level OVCFs (January 2021–January 2023) were randomized to PCKP (n = 67) or UPKP (n = 72), with ≥24-month follow-up. Outcomes included the visual analog score (VAS), Oswestry dysfunction index (ODI), cement distribution (type I–V classification), leakage rate, and re-fracture incidence. Anti-osteoporosis compliance was analyzed for its impact on efficacy.

**Results:**

Both groups showed significant postoperative improvement in VAS and ODI (*p* < 0.05). PCKP achieved superior early pain relief (VAS: 1.99 ± 0.77 vs. 3.47 ± 0.50; ODI: 27.07 ± 1.78 vs. 35.33 ± 3.12 at 2 days, *p* < 0.001), a higher cement distribution excellence rate (91.04% vs. 76.39%), and a lower leakage rate (10.45% vs. 26.39%). However, outcomes converged from 3 months onward (*p* > 0.05), with no significant differences in re-fracture (11.94% vs. 15.28%) or anti-osteoporosis compliance (28.36% vs. 33.33%). Subgroup analysis revealed that PCKP provided more symmetric cement distribution (*p* = 0.02) and a 21% lower leakage risk in moderate-to-severe OVCFs (Genant 2–3).

**Conclusion:**

PCKP enhances early biomechanical stability in moderate-to-severe OVCFs through optimized cement dispersion, while long-term efficacy relies on standardized anti-osteoporosis therapy. A stepwise decision model (“PCKP for Genant 2–3, UPKP for mild cases”), combined with a “vertebral augmentation–bone metabolism modulation–behavioral intervention” strategy, is recommended.

## 1 Background

Osteoporotic vertebral compression fractures (OVCFs) due to osteoporosis have emerged as a significant and prevalent public health concern in the contemporary context of an aging population (Weber et al., 2024). These fractures have become a substantial source of pain and dysfunction in older adults (Al Taha et al., 2024). The impact of OVCFs on patients’ quality of life, recovery of social function, and the associated socioeconomic and health insurance burden is considerable. A substantial body of research has demonstrated that vertebroplasty significantly reduces the risk of mortality associated with osteoporotic vertebral compression fractures, particularly during the early post-fracture period (You et al., 2025). Among these, percutaneous vertebral kyphoplasty has gained widespread adoption due to its efficacy in improving kyphosis and reducing leakage rates.

The current state of the literature indicates that unilateral and bilateral approaches to percutaneous kyphoplasty (PKP) can achieve similar efficacy (Sun N. et al., 2024; Cao et al., 2024). However, each approach is associated with significant technical challenges. Unilateral PKP requires a larger puncture angle, which is prone to nerve injury, while bilateral PKP prolongs operative time and increases radiation exposure (Wang et al., 2021; Tao et al., 2024). In recent years, the emergence of percutaneous curved kyphoplasty (PCKP) has shown significant advantages in improving cement distribution and reducing leakage rates through improved instrument design (Hu et al., 2024). However, a subsequent review and analysis revealed significant limitations in the available evidence. The follow-up period is mostly shorter than 1 year, there is a lack of mid- and long-term efficacy data (Shi et al., 2023), and the applicability to patients with moderate-to-severe fractures (vertebral body height loss >40%) has not been elucidated (Lv et al., 2023).

In light of the aforementioned research limitations, the present study was conceived as a prospective randomized controlled trial with two core research questions in mind: first, the efficacy of PCKP would be compared with that of UPKP in the treatment of single-segment OVCFs, with a focus on intermediate- and long-term (≥2 years) clinical outcomes, including pain relief (VAS score), functional recovery (ODI index), and complication rates. Second, the degree of vertebral compression on the efficacy of PCKP would be explored, with the aim of establishing quantitative criteria for patient screening. The results of this study will provide a high-level evidence-based basis for optimizing minimally invasive treatment strategies for OVCFs. This will be of great clinical significance in reducing medical costs and improving the quality of life of elderly patients.

## 2 Subjects and methods

### 2.1 Study design

This study was a prospective randomized controlled trial (approved by the Ethics Committee of the First People’s Hospital of Zigong), and the study subjects were patients admitted to the Department of Orthopedics of the First People’s Hospital of Zigong from January 2021 to January 2023.

### 2.2 Inclusion and exclusion criteria

Inclusion criteria:

- Diagnosis of acute single-segment OVCFs by imaging (X-ray, CT, MRI, or bone scan if contraindicated).
- Failure of conservative treatment (≥2 weeks) or proven Kummel disease with persistent pain or dysfunction requiring improved quality of life.
- Able to tolerate surgery and cooperate with follow-up.
- Those who obtained complete follow-up after surgery.

Exclusion criteria:

• Multi-segmental OVCFs.
• Patients with serious comorbidities (e.g., uncontrolled cardiopulmonary disease) who cannot tolerate surgery.
• Patients and family members who refused surgery and requested conservative treatment.
• Missing visits or refusing to participate in the study.

### 2.3 Randomization group

A single-blind randomization scheme was used to group patients based on computer-generated random numbers. An independent third party grouped patients with eligible OVCFs in either the PCKP group (67 patients) or the unilateral percutaneous kyphoplasty (UPKP) group (72 patients). The baseline characteristics of patients in the two groups, including vertebral compression severity (using the Genant semi-quantitative grading, defined as mild for Genant grades 0–1, moderate for grade 2, and severe for grade 3), were balanced and comparable.

### 2.4 Surgical procedures

All surgeries were performed by experienced spine surgeons from the same surgical team who are skilled in vertebral body strengthening techniques.

Surgical instruments and materials: PCKP and UPKP surgical instrument systems were provided by Shanghai Kellett Medical Technology Co. Bone cements were all made of polymethylmethacrylate (PMMA) produced by Mendec Spine Resin, Italy.

A total of 197 patients with single-segment OVCFs were initially enrolled in this study, and after screening with strict inclusion and exclusion criteria, 171 patients met the conditions, 27 were lost during data follow-up, and 5 were withdrawn from the study due to death, resulting in a complete collection of data from 139 patients with acute single-segment thoracolumbar OVCFs, including 55 male and 84 female individuals. All of them were low- or no-energy-caused injuries, and the distribution of fractured vertebrae was as follows: 5 cases of T6, 7 cases of T7, 9 cases of T8, 9 cases of T9, 12 cases of T10, 14 cases of T11, 19 cases of T12, 21 cases of L1, 16 cases of L2, 11 cases of L3, 9 cases of L4, and 7 cases of L5, of which the thoracolumbar combined segment (T10–L2) accounted for 58.99% (82/139).

### 2.5 Key points of the surgical operation

#### 2.5.1 Preoperative preparation and anesthesia

• The patient is positioned in the prone position, and cardiac monitoring, oxygenation, and open intravenous access are initiated.
• A local infiltration anesthetic (a mixture of lidocaine and ropivacaine) is administered.
• G-arm X-ray fluoroscopy is used to locate the target vertebral body, which is then disinfected and covered with towels.

#### 2.5.2 Operation steps

PCKP: Unilateral puncture, curved bone drill along the curved channel across the midline, and deflectable balloon dilatation to cover the inner edge of the contralateral pedicle, with no need to repeatedly adjust the puncture angle.

UPKP: Unilateral puncture, linear bone drill, and linear balloon dilatation to the middle of the vertebral body, requiring multiple adjustments of the puncture angle and pressure to expand diffusion.

#### 2.5.3 Postoperative care and follow-up procedures

• Bone cement should be prepared to a consistency analogous to toothpaste and then injected slowly, with cessation when the posterior one-quarter of the vertebral body is reached or when leakage is detected on X-ray monitoring.
• Postoperative cardiac monitoring and oxygenation are performed for 24 h, and the patient is discharged from the hospital at 72 h, with outpatient follow-up at 3, 12, and 24 months.

Standardized anti-osteoporosis treatment, comprising calcium and vitamin D3 supplementation, is administered in conjunction with zoledronic acid or teriparatide as indicated. Blood and urine calcium monitoring is conducted every 3 months, and bone density is assessed annually. Imaging follow-up involves repeating X-rays 2 days post-surgery to evaluate cement distribution, with CT scans utilized for symptomatic leakage.

Long-term follow-up involves reviewing functional and imaging parameters at 3, 12, and 24 months postoperatively.

The primary observational indices encompass the following: (1) pain and functional maintenance: visual analog score (VAS), Oswestry dysfunction index (ODI), and anti-osteoporosis compliance rate (preoperative, postoperative 2 days, 3 months, 12 months, and 24 months); (2) imaging indices: type of distribution of the bone cement (classified as I–V type, with IV/V type considered maldistributed) and the amount of bone cement injected; and (3) complications (i.e., cement leakage rate and re-fracture rate).

The statistical analysis method used all data collected using SPSS 20.0 software. The measurement data were expressed as (±S), and the t-test for two independent samples was used for comparison of measurement data within and between groups. The χ^2^ test was used for comparison of count data, and the non-parametric test was used for grade data. The significance level was set at α = 0.05, and *p* < 0.05 was considered statistically significant.

## 3 Results

The surgical procedure was successfully completed, and postoperative follow-up data were obtained for 139 patients with OVCFs for a minimum of 24 months, with a mean follow-up period of (24.98 ± 0.74) months. The age range of patients with OVCFs in the PCKP group was 58–99 years, while the age range of patients with OVCFs in the UPKP group was 57–96 years. A meticulous examination revealed no statistically significant disparities between the two groups with respect to age, gender, BMD T-value, duration of pain, and degree of compression (*p* > 0.05). Refer to Table 1 for comprehensive details.

**TABLE 1:** Comparison of general information of the two groups of patients.

Data		PCKP (n = 67)	UPKP (n = 72)	t/χ^2^-value	*p*-value
Age ( $\bar{x}$ ±s, years)		78.42 ± 12.68	77.26 ± 13.06	0.53	0.60
Gender	Male	24 (35.82)	23 (31.94)	0.03	0.86
	Female	43 (64.18)	49 (68.06)		
Bone density T-value ( $\bar{x}$ ±s, SD)		−(3.68 ± 0.83)	−(3.96 ± 0.82)	−1.29	0.48
Duration of pain ( $\bar{x}$ ±s, days)		6.68 ± 3.67	7.38 ± 3.72	−0.72	0.85
Grading of compression (Genant semi-quantitative method)	Mild (Genant 0-1)	26	30	1.09	0.58
	Moderate (Genant 2)	22	27		
	Severe (Genant 3)	19	15		

As illustrated in Table 2, a comparison of preoperative and postoperative scores at various time points within the PCKP and UPKP groups reveals significant differences in VAS and ODI scores (*p* < 0.05). However, there is no statistically significant difference in the comparison of VAS and ODI scores between the two groups at 2 days postoperatively (*p* < 0.05); and there was also no significant difference in the comparison of VAS and ODI scores between the two groups at preoperatively and at 3, 12, and 24 months postoperatively (*p* > 0.05).

**TABLE 2:** Comparison of preoperative and postoperative VAS and ODI between the two groups of patients.

Group		PCKP (n = 67)	UPKP (n = 72)	*t*-value	*p*-value
VAS score ( $\bar{x}$ ±s, points)	Preoperative	7.18 ± 1.74	7.68 ± 1.73	−1.70	0.09
	2 days postoperatively	1.99 ± 0.77	3.47 ± 0.50	−13.59	0.000
	3 months postoperatively	1.60 ± 1.05	1.64 ± 1.18	−0.22	0.83
	12 months postoperatively	0.87 ± 0.80	0.89 ± 0.85	−0.17	0.85
	24 months postoperatively	0.69 ± 0.63	0.76 ± 0.74	−0.66	0.51
ODI index( $\bar{x}$ ±s, points)	Preoperative	81.12 ± 6.38	81.60 ± 5.75	−0.46	0.64
	2 days postoperatively	27.07 ± 1.78	35.33 ± 3.12	−19.00	0.000
	3 months postoperatively	24.79 ± 3.01	25.42 ± 3.05	−1.22	0.23
	12 months postoperatively	23.37 ± 2.47	24.25 ± 2.96	−1.89	0.06
	24 months postoperatively	22.84 ± 2.04	23.29 ± 3.78	−0.88	0.38

*p* < 0.05 for intra-group VAS scores, ODI index after 2 days, 3 months, and 12 months compared with the preoperative period in both groups.

Table 3 shows that there is no significant difference in the comparison of the anti-osteoporosis compliance rate and incidence of re-fracture between the two groups of patients in PCKP and UPKP (*p* > 0.05), while there is a significant difference in the comparison of the dose of bone cement injected, the rate of excellent distribution of bone cement, and the rate of bone cement leakage between the two groups of patients (*p* < 0.05).

**TABLE 3:** Comparison of surgical and postoperative data between the two groups of patients.

Group	Bone cement dose ( $\bar{x}$ ±s, ml)	Excellent rate of bone cement distribution (case, %)	Bone cement leakage rate (case, %)	Anti-osteoporosis treatment compliance rate (case, %)	Re-fracture rate (case, %)
PCKP	4.75 ± 1.28	61 (91.04%)	7 (10.45%)	19 (28.36%)	8 (11.94%)
UPKP	3.25 ± 1.12	55 (76.39%)	19 (26.39%)	25 (33.33%)	11 (15.28%)
t/χ^2^-value	7.33	5.40	5.80	0.65	0.33
P-value	0.000	0.02	0.02	0.42	0.57

As illustrated in Table 4, there is no statistically significant difference (*p* > 0.05) in the comparison of cement distribution and leakage between the two patient groups with PCKP and UPKP at varying compression levels (mild, moderate, and severe). The table indicates that in cases of moderate and severe compression fractures, PCKP demonstrates a higher rate of cement distribution and leakage.

**TABLE 4:** Comparison of cementation profiles of patients with different degrees of compression in the two groups.

Group	Bone cement distribution			Bone cement leakage		
	Mild	Moderate	Severe	Mild	Moderate	Severe
PCKP	2	2	2	2	3	2
UPKP	5	6	6	5	8	6
χ^2^-value	0.03			0.03		
P-value	0.98			0.99		

The adverse reaction group (PCKP) exhibited seven cases of cement leakage, including two cases of intervertebral space leakage, four cases of paravertebral soft tissue leakage, and one case of intravertebral canal leakage. The UPKP group demonstrated 19 cases of cement leakage, including four cases of intervertebral space leakage, fourteen cases of paravertebral soft tissue leakage, and one case of intravertebral canal leakage. Intravertebral canal leakage occurred in both groups; however, the cement infusion was immediately halted upon discovery by G-arm fluoroscopy, and no patients exhibited signs of spinal cord or nerve damage postoperatively; therefore, no further treatment was administered. No cases of serious complications, including bone cement allergy, bone cement pulmonary embolism, or bone cement leakage resulting in clinical symptoms, were observed. The surgical procedure was successfully completed, and a comprehensive postoperative follow-up was conducted.

## 4 Discussion

In this study, a prospective randomized controlled comparison of the medium- and long-term clinical efficacy of PCKP and UPKP in the treatment of single-segment OVCF was conducted. The results of this study revealed significant advantages of PCKP in biomechanical optimization, pain alleviation, and the reduction of cement leakage. Furthermore, the study’s findings contributed to a more profound understanding of the postoperative re-fracture mechanism. This was achieved by examining the postoperative anti-osteoporosis compliance rate and re-fracture incidence. Additionally, the study underscored the significance of postoperative anti-osteoporosis treatment and provided a substantial evidence-based foundation for individualized treatment strategy for OVCF.

### 4.1 Relationship between early analgesic advantage and bone cement distribution

In the present study, we demonstrated that PCKP exhibited a significant advantage in terms of early efficacy, manifesting as rapid pain relief at 2 days postoperatively. This was evidenced by a 42.65% improvement in the VAS score compared with UPKP and a 23.38% improvement in the ODI index compared with UPKP. Lv et al. (2023) observed a positive correlation between early pain relief after vertebroplasty and symmetrical bone cement distribution. This observation underscores the significance of adequate bone cement distribution and filling in determining the efficacy of vertebroplasty treatment. A comparative analysis of the baseline data from the two patient groups included in this study, operated on by the same surgeon, revealed no statistically significant differences (p > 0.5). However, the superiority of PCKP in terms of pain management appears to be associated with its capacity to achieve adequate cement filling within the vertebral body using a curved bone drill, thereby implementing the “stress rebalance theory.” First, PCKP creates a continuous stress gradient by loosening the dense trabeculae through the curved bone drill, thus overcoming the mechanical limitations of the traditional linear puncture path of UPKP. Second, the Kummel-like cavity generated by the curved balloon expansion of PCKP has a synergistic effect on the hydrodynamic characteristics of the cement under low-pressure perfusion, which can significantly reduce the resistance to cement injection and achieve synergy between low-pressure perfusion and the microarchitecture of cancellous bone, effectively circumventing the “rebalancing theory” of UPKP. This matching approach significantly reduces resistance to cement injection, achieving a high bone cement filling rate (91.04% vs. 76.39%) and a more satisfactory of early pain relief effect. Furthermore, Sun Y. et al. (2024) observed that when the intravertebral cement filling rate ranged from 0.4 to 0.6, a favorable equilibrium was attained between the mitigation of complications and the promotion of favorable treatment outcomes. This finding is further supported by the significant early pain relief observed, which suggests that the uniform distribution of bone cement can more effectively create microfractures in the vertebral body, achieve anatomical–mechanical two-dimensional reconstruction, optimize the vertebral load transfer path, and thus reduce local stress concentration to achieve biomechanical stabilization, which results in better early pain relief. This also verifies the possible mechanism of better early pain relief in PCKP, i.e., the “cement dispersion-pain relief” correlation. This is highly consistent with the findings of Yuntao et al. (2025); that is, better cement diffusion leads to better early pain relief. Therefore, Cheng and Xie (2024) suggested that a unilateral approach is recommended for level I OVCFs, whereas a bilateral approach is preferred for levels II and III OVCFs. The purpose of this approach is to achieve better vertebral height regression and cement filling diffusion. In a related study, Jia et al. (2025) categorized the cement distribution on postoperative radiographs into four types based on fracture bone marrow edema area (FBMEA) in preoperative MRIs of patients with OVCFs. This finding further indicates that appropriate bone cement use is beneficial for functional improvement, short-term pain relief, and reduction in the vertebral body recollapse rate. This underscores the necessity of closely monitoring the status of bone cement and its effectiveness in filling the vertebral body during treatment, with the aim of ensuring treatment efficacy and mitigating complications (Bian et al., 2024). In this study, we demonstrated that in the absence of spinal cord and nerve root injuries, there was no correlation between early pain relief and cement leakage after vertebroplasty. However, the medium- and long-term efficacy of the treatment may have a strong correlation with cement leakage, especially in cases of intervertebral cement leakage. This may be due to the deterioration of the local biomechanical environment, which increases the risk of secondary proximal vertebral fracture (Xie et al., 2025). In this study, the amount of cement used was significantly higher in the PCKP group (4.75 ± 1.28 mL vs. 3.25 ± 1.12 mL), yet the leakage rate was still significantly lower than that of UPKP (10.45% vs. 26.39%), and the difference was even more pronounced in moderate and severe cases (21.13% lower leakage rate). We have determined that UPKP utilizes a linear bone drill and a balloon with the puncture biased to one side. To achieve symmetric dispersion of the bone cement during the operation, it is necessary to rely on the high-pressure injection of the bone cement to compensate for the dispersion defects. This results in a significant increase in the risk of cement leakage with an increase in the amount of cement injected. In contrast, PCKP utilizes a distinct curved bone drill, a deflectable balloon, and directional cement infusion instruments, enabling the creation of the bony cavity and low-pressure infusion. This approach increases the dose of bone cement and reduces the leakage rate to a certain extent. Concurrently, this phenomenon indicates that the risk of cement leakage is not solely associated with the dose but rather with the congruence between the perfusion pattern and the bone microstructure.

### 4.2 Relationship between long-term efficacy convergence and anti-osteoporosis

Despite the significant pain relief advantage of PCKP in the early postoperative period (2 days postoperatively) in the present study, the VAS scores and functional recovery (ODI) effects converged between the two groups of patients at 3, 12, and 24 months postoperatively (*p* > 0.5). The failure of PCKP’s early pain relief advantage to be sustained over time is a significant discrepancy with the structure of some of the current studies (Fu et al., 2023; Huang et al., 2024). Lindsay et al. (2001) showed that OVCF patients had a 20% risk of subsequent fracture within 1 year once they became symptomatic. A comprehensive analysis of numerous studies (Li et al., 2023; Pan et al., 2023) revealed that satisfactory cement distribution in vertebroplasty can achieve enhanced biomechanics, improved clinical outcomes, and reduced re-fracture rates. However, our current study demonstrated that there was no statistically significant difference in the postoperative vertebral re-fracture rates between the two patient groups. We conducted a thorough investigation to elucidate the reasons behind the early benefits of PCKP not being sustained. We hypothesize that this phenomenon may be attributable to the following factors. First, the study is limited by its single-center design and small sample size, which may lead to a statistically significant increase in the risk of class II error. This is particularly salient given that re-fracture is a relatively low-probability event that necessitates verification by additional centers and large-scale cohorts. Second, bone metabolic imbalance is a fundamental driver of re-fracture, and the current clinical focus of both patients with OVCFs and spine surgeons may be overly concentrated on treating the fracture itself as a complication and trivializing the etiologic treatment of osteoporosis. Numerous studies have established that anti-osteoporosis treatment is a protective factor against vertebral re-fracture (Yang et al., 2025; Lu et al., 2024). Furthermore, the incidence of vertebral re-fracture correlates with the patient’s own BMD and postoperative anti-osteoporosis (Prost et al., 2021; Takahashi et al., 2019). Finally, the treatment of OVCFs is a long-term, chronic process, which emphasizes the need to prioritize patient compliance with standardized anti-osteoporosis treatment and to enhance compliance to the greatest extent possible to ensure therapeutic efficacy. In a real-world study by Zou et al. (2024), it was observed that the percentage of patients using anti-osteoporosis drugs 6 months after vertebral body strengthening was low, with only approximately 7% using denosumab and 13%–15% using oral bisphosphonates. The persistent nature of osteoporosis engenders an elevated risk of vertebral re-fracture.

It is imperative to acknowledge that even with standardized anti-osteoporosis treatment, the correction of bone metabolism in elderly patients with OVCF necessitates a protracted recovery period, accompanied by a “drug response–bone metabolism repair” refractory period. Tang et al. (2024) demonstrated that there is a gradual and persistent loss of height of cement-reinforced vertebrae in the postoperative period. It is important to note that all current anti-osteoporosis therapeutic agents work by promoting osteogenesis or decreasing osteolysis and reducing fracture occurrence (LeBoff et al., 2022). All anti-fracture medications treat but do not cure osteoporosis. Once the medication is discontinued, bone deterioration returns sooner or later, and the risks of osteoporosis and fracture remain even normal BMD has been achieved on follow-up assessment after anti-fracture therapy. In addition, Reid and Billington (2022) demonstrated that anabolic drugs exhibit superior anti-fracture efficacy and more substantial increases in BMD compared to anti-bone resorption drugs. Nevertheless, the effects of anabolic drugs are transient, necessitating a transition to anti-bone resorption drugs. Consequently, to reduce the re-fracture rate in our clinic, it is essential to implement a multifaceted approach that incorporates anti-osteoporotic drugs (which promote osteogenesis and inhibit osteoblasts), adequate intake of calcium and vitamin D (the basic medication), avoidance of smoking and excessive alcohol intake, weight-bearing and resistance training, and the prevention of falls. Therefore, long-term postoperative maintenance of outcomes in patients with OVCF may require greater emphasis on anti-osteoporotic therapy to reduce the risk of re-fracture. Consequently, this study challenges the conventional “cement distribution-centered” approach to OVCF treatment and underscores the imperative for a multifaceted strategy that incorporates early mechanical stabilization and prolonged bone metabolism regulation.

### 4.3 Surgical selection and comprehensive postoperative management strategies for moderate-to-severe OVCF

This study demonstrated that, for moderate and severe OVCF cases (Genant grade 2–3), PCKP offers clear advantages by enhancing the efficacy of cement distribution (91.04% vs. 76.39%) and mitigating the risk of leakage (10.45% vs. 26.39%). In contrast, for mild OVCF cases (Genant 0–1), UPKP remains a viable option due to its relative ease of operation. However, it is crucial to note that the risk of leakage due to high-pressure perfusion should be carefully considered. The “two-level decision model” developed based on the Genant classification utilizes the “vertebral compression degree” as the primary index for determining the surgical approach, providing a significant foundation for clinical decision-making. Early satisfactory clinical outcomes can be attained.

In addition, to achieve a satisfactory long-term clinical outcome, the postoperative management of PCKP and UPKP should be based on a strategy of “treating both the symptoms and the root cause,” with short-term relief of acute pain through vertebroplasty and long-term stepwise anti-osteoporosis (calcium supplementation, promotion of osteogenesis, and inhibition of osteoclastogenesis). A stepwise intervention is recommended, including restoration of vertebral height, early pain relief, and restoration of function through PCKP/UPKP in the acute phase; maintenance of calcium + vitamin D + bisphosphonate/teriparatide/romosozumab combination therapy for 24 months postoperatively; detection of dynamic changes in bone metabolism through bone mineral density; and integration of fall prevention and rehabilitation to form a “surgery-drug-behavioral” strategy, also known as the “surgery-drug-behavior” trinity management model (LeBoff et al., 2022).

### 4.4 Study limitations and future directions

This study is not without its limitations. The single-center design may introduce selection bias, the 24-month follow-up makes it difficult to assess ultra-long-term (>5 years) biomechanical changes, and bone metabolism markers (e.g., β-CTX and P1NP) and further quantification of treatment adherence were not included. Future studies must conduct multicenter RCTs to reduce selection bias and improve the reliability and generalizability of the results. The development of a prediction model integrating finite element analysis and “OVCF classification–procedure matching” will facilitate more precise procedure selection and enhance therapeutic efficacy. Furthermore, investigating the synergistic mechanisms between anti-osteoporosis medications and bone cement materials has the potential to yield novel therapeutic strategies and provide a theoretical foundation for the treatment of OVCFs.

## 5 Conclusion

The advent of curved bone drilling technology and low-pressure perfusion mode has catalyzed PCKP’s transition from an “empirical operation” to a “precise decision-making” paradigm in the management of moderate-to-severe OVCF. The value of this innovation is multifaceted, as evidenced by the reduction in the leakage rate and early pain control. Moreover, it has led to a fundamental paradigm shift in the treatment of OVCF, moving from a single focus on local biomechanical repair to a systematic management of mechanical stabilization, bone metabolism regulation, and behavioral intervention. The decision tree of “vertebral compression grading–operative selection” established in this study provides a new paradigm for individualized treatment and also provides high-level evidence to support the updating of global OVCF treatment guidelines.

## Data Availability

The raw data supporting the conclusions of this article will be made available by the authors upon reasonable request.

## References

[B1] Al Taha K. Lauper N. Bauer D. E. Tsoupras A. Tessitore E. Biver E. (2024). Multidisciplinary and coordinated management of osteoporotic vertebral compression fractures: current state of the art. J. Clin. Med. 13 (4), 930. 10.3390/jcm13040930 38398244 PMC10889683

[B2] Bian C. Gu H. Chen G. Cheng X. Huang Z. Xu J. (2024). A retrospective study of 91 patients treated with percutaneous kyphoplasty for mild osteoporotic vertebral compression fractures and a new evaluation scale of shape and filling effect of cement. World Neurosurg. 186, e134–e141. 10.1016/j.wneu.2024.03.089 38522788

[B3] Cao D. H. Gu W. B. Zhao H. Y. Hu J. L. Yuan H. F. (2024). Advantages of unilateral percutaneous kyphoplasty for osteoporotic vertebral compression fractures-a systematic review and meta-analysis. Arch. Osteoporos. 19 (1), 38. 10.1007/s11657-024-01400-8 38750277

[B4] Cheng Y. Xie X. (2024). Therapeutic effects of single versus bilateral approaches for percutaneous kyphoplasty in osteoporotic compression fractures. Asian J. Surg. 47 (5), 2272–2273. 10.1016/j.asjsur.2024.01.149 38341374

[B5] Fu X. Li Y. M. Tian P. Xu G. J. Li Z. J. (2023). Comparison between unilateral curved and bilateral straight percutaneous vertebral augmentation in the treatment of osteoporotic vertebral compression fractures: a meta-analysis. Jt. Dis. Relat. Surg. 34 (2), 237–244. 10.52312/jdrs.2023.967 37462625 PMC10367155

[B6] Hu B. Zhang X. Yang Q. Zheng C. Mhammad A. S. Hao M. (2024). Comparison of the efficacy and safety of vertebroplasty with different pedicle approaches for osteoporotic vertebral. Eur. Spine J. 33 (8), 3191–3212. 10.1007/s00586-024-08240-7 38965088

[B7] Huang Y. Liu Y. Zhong F. Zhou X. Huang S. Huang C. (2024). Percutaneous curved vertebroplasty Versus unilateral percutaneous vertebroplasty for osteoporotic vertebral compression fractures: a systematic review and meta-analysis. World Neurosurg. 181, 29–37. 10.1016/j.wneu.2023.10.035 37839572

[B8] Jia R. Li D. He P. Wang X. Q. Zhang Y. Bai J. (2025). Impact of cement distribution on the efficacy of percutaneous vertebral augmentation for osteoporotic fractures: assessment with an MRI-based reference marker. J. Bone Jt. Surg. Am. 107 (2), 196–207. 10.2106/JBJS.23.01289 39812726

[B9] LeBoff M. S. Greenspan S. L. Insogna K. L. Lewiecki E. M. Saag K. G. Singer A. J. (2022). The clinician’s guide to prevention and treatment of osteoporosis. Osteoporos. Int. 33 (10), 2049–2102. 10.1007/s00198-021-05900-y 35478046 PMC9546973

[B10] Li K. C. Hsieh C. H. Liao T. H. Cheng B. H. (2023). A novel classification of cement distribution patterns based on plain radiographs associated with cement filling rate and relevance to the clinical results of unipedicle vertebroplasty. Eur. Spine J. 32 (1), 101–109. 10.1007/s00586-022-07412-7 36220958

[B11] Lindsay R. Silverman S. L. Cooper C. Hanley D. A. Barton I. Broy S. B. (2001). Risk of new vertebral fracture in the year following a fracture. JAMA 285 (3), 320–323. 10.1001/jama.285.3.320 11176842

[B12] Lu Y. Cai X. Shen J. Luo R. (2024). Development and validation of a prediction model for vertebral recompression and adjacent vertebral fracture after kyphoplasty in geriatric patients. Eur. Spine J. 10.1007/s00586-024-08485-2 39245779

[B13] Lv Z. Chen Z. Chen H. Wang J. Han Y. Li X. (2023). Percutaneous curved vertebroplasty Versus unipedicular approach vertebroplasty for acute osteoporotic vertebral compression fractures: a randomized controlled trial. Spine (Phila Pa 1976) 48 (8), 552–558. 10.1097/BRS.0000000000004593 36763817

[B14] Pan Z. Zhou Q. Yang M. Deng L. Hu X. Lv N. (2023). Effects of distribution of bone cement on clinical efficacy and secondary fracture after percutaneous kyphoplasty for osteoporotic vertebral compression fractures. Front. Surg. 9, 1054995. 10.3389/fsurg.2022.1054995 36684222 PMC9852057

[B15] Prost S. Pesenti S. Fuentes S. Tropiano P. Blondel B. (2021). Treatment of osteoporotic vertebral fractures. Orthop. Traumatol. Surg. Res. 107 (1S), 102779. 10.1016/j.otsr.2020.102779 33321233

[B16] Reid I. R. Billington E. O. (2022). Drug therapy for osteoporosis in older adults. Lancet. 399 (10329), 1080–1092. 10.1016/S0140-6736(21)02646-5 35279261

[B17] Shi X. Li P. Li J. Bao C. Xiang J. Lu Y. (2023). Comparative evaluation of an innovative deflectable percutaneous kyphoplasty versus conventional bilateral percutaneous kyphoplasty for osteoporotic vertebral compression fractures: a prospective, randomized and controlled trial. Spine J. 23 (4), 585–598. 10.1016/j.spinee.2022.12.012 36563860

[B18] Sun N. Zhang Y. Xie D. Chen Y. Liu Y. (2024). Enhancing percutaneous kyphoplasty efficacy in elderly osteoporotic fractures through optimal cement filling ratio. Front. Endocrinol. (Lausanne) 15, 1359550. 10.3389/fendo.2024.1359550 38800478 PMC11116659

[B19] Sun Y. Li X. Ma S. Chong H. Cai T. C. Li K. M. (2024). Comparison of the efficacy and safety of unilateral and bilateral approach kyphoplasty in the treatment of osteoporotic vertebral compression fractures: a meta-analysis. Jt. Dis. Relat. Surg. 35 (3), 491–503. 10.52312/jdrs.2024.1701 39189557 PMC11411882

[B20] Takahashi S. Hoshino M. Yasuda H. Hori Y. Ohyama S. Terai H. (2019). Development of a scoring system for predicting adjacent vertebral fracture after balloon kyphoplasty. Spine J. 19 (7), 1194–1201. 10.1016/j.spinee.2019.02.013 30831317

[B21] Tang Z. Q. He S. B. Yu D. Y. Luo H. M. Xing X. H. Zhou Y. W. (2024). Factors influencing further vertebral height loss following percutaneous vertebroplasty in osteoporotic vertebral compression fractures: a 1-year follow-up study. World J. Clin. Cases 12 (21), 4609–4617. 10.12998/wjcc.v12.i21.4609 39070819 PMC11235515

[B22] Tao H. Huang Z. Shao S. Yang R. Yang K. Zhang Y. (2024). Comparison of robot-assisted and fluoroscopy-assisted percutaneous kyphoplasty for bone cement distribution and clinical efficacy. Pain Physician 27 (8), E953–E963. 39621996

[B23] Wang C. Zhang Y. Chen W. Guo K. J. Feng S. (2021). Comparison of percutaneous curved kyphoplasty and bilateral percutaneous kyphoplasty in osteoporotic vertebral compression fractures: a randomized controlled trial. BMC Musculoskelet. Disord. 22 (1), 588. 10.1186/s12891-021-04469-1 34174859 PMC8234738

[B24] Weber A. Vercoulen T. F. G. Jacobs E. Buizer A. T. Bours S. P. G. van den Bergh J. P. (2024). Disparities in management of symptomatic osteoporotic vertebral compression fractures: a nationwide multidisciplinary survey. Arch. Osteoporos. 19, 101. 10.1007/s11657-024-01454-8 39441383 PMC11499376

[B25] Xie S. Cui L. Wang C. Liu H. Ye Y. Gong S. (2025). Contact between leaked cement and adjacent vertebral endplate induces a greater risk of adjacent vertebral fracture with vertebral bone cement augmentation biomechanically. Spine J. 25 (2), 324–336. 10.1016/j.spinee.2024.09.021 39343240

[B26] You Z. Wu K. Jiang Y. Chen J. (2025). Effect of vertebral kyphoplasty versus vertebroplasty on pain and indicators of imaging parameters of the injured vertebrae in patients with osteoporotic vertebral compression fractures: a meta-analysis. J. Orthop. Surg. Res. 20 (1), 199. 10.1186/s13018-025-05621-6 40001072 PMC11863920

[B27] Yang W. Zou K. Lin X. Yang Y. Chen T. Wu X. (2025). Risk factors for new vertebral fractures after percutaneous vertebroplasty or percutaneous kyphoplasty in the treatment of osteoporotic vertebral compression fractures. Front. Med. (Lausanne) 12, 1514894. 10.3389/fmed.2025.1514894 39911860 PMC11794209

[B28] Yuntao L. Haibier A. Kayierhan A. Liang M. Abudukelimu Y. Aximu A. (2025). Clinical effect analysis of unilateral percutaneous vertebral cement distribution in the repair of osteoporotic thoracolumbar vertebral compression fractures. BMC Surg. 25 (1), 90. 10.1186/s12893-025-02820-0 40045301 PMC11881425

[B29] Zou J. Zhang Y. Niu J. Song D. Huang Z. Li Z. (2024). A real-world study of denosumab for reducing refracture risk after percutaneous vertebral augmentation. Orthop. Surg. 16 (8), 1849–1860. 10.1111/os.14087 38952145 PMC11293904

